# A case series of dermatomyositis following SARS-CoV-2 vaccination

**DOI:** 10.3389/fmed.2022.1013378

**Published:** 2022-11-07

**Authors:** Airiss R. Chan, Jan Willem Cohen Tervaert, Desiree Redmond, Elaine Yacyshyn, Giovanni Ferrara, Peter M. Hwang, Mohamed Osman, Robert Gniadecki

**Affiliations:** ^1^Division of Dermatology, University of Alberta, Edmonton, AB, Canada; ^2^Division of Rheumatology, University of Alberta, Edmonton, AB, Canada; ^3^Division of Pulmonary Medicine, University of Alberta, Edmonton, AB, Canada; ^4^Division of General Internal Medicine, University of Alberta, Edmonton, AB, Canada

**Keywords:** vaccine, dermatomyositis, SARS-CoV-2, exacerbation, TLR7, molecular mimicry

## Abstract

**Background/Objective:**

The most significant adverse events following SARS-CoV-2 vaccination are myocarditis and pericarditis. Myositis and dermatomyositis have been reported following SARS-CoV-2 infection, but vaccine-induced dermatomyositis (DM) has not been reported. Our case series aimed to characterize new onset dermatomyositis or disease-related flares following SARS-CoV-2 vaccination.

**Materials and methods:**

A total of 53 patients from our institution with a new or pre-existing diagnosis of DM were recruited and consented. Phone interviews were conducted to obtain vaccination status and symptoms following vaccination. Electronic medical records were reviewed to extract age, sex, autoantibody profiles, comorbidities, immunomodulatory therapies, creatine kinase (CK) values, and SARS-CoV-2 vaccination dates from the provincial vaccination registry. For patients who reported disease flares, records were reviewed for the onset and nature of symptoms, extent of organ involvement and changes in immunomodulation.

**Results:**

On average, patients received 2.62 vaccine doses (range 1–3 doses). A total of 3 of 51 patients (5.88%) experienced dermatomyositis symptoms following vaccination. Two patients were newly diagnosed with dermatomyositis, one requiring hospitalization. Reported symptom onset following vaccination ranged from 1 to 30 days. Of note, all of these patients had normal CK values, even though there was muscle biopsy-confirmed myositis in one patient. Eight patients in the cohort (15.1%) had asymptomatic CK elevation (<1.5 X ULN).

**Conclusion:**

New onset dermatomyositis or flare up of pre-existing dermatomyositis may be a rare complication in SARS-CoV-2 vaccination although no studies can support a true correlation. Several pathophysiologic mechanisms are proposed.

## Introduction

As of May 2022, 84.9% of the population of Canada (32.4 million) received at least one dose of the vaccine against severe acute respiratory syndrome coronavirus 2 (SARS-CoV-2) (1). Of the 114,108 adverse events reported to date as of 29 April 2022, myocarditis and pericarditis comprise 0.93% of cases, the most common autoimmune complications (2, 3). Although most cases of myocarditis have been classified as mild, skeletal muscle inflammation may also ensue, resulting in significant sequelae (4).

Dermatomyositis (DM) is associated with systemic inflammation of the skin and muscles. Cases of DM in the context of COVID-19 infection have been previously reported in the literature (5–13) and DM following hepatitis B, influenza, tetanus, and Bacillus Calmette-Guérin (BCG) vaccines have also been reported. However, DM developing after SARS-CoV-2 vaccination has not yet been reported.

To determine the risk of DM flare after SARS-CoV-2 vaccines, we performed a single-institution retrospective case series compromising all patients with the diagnosis of DM. We report two cases of new-onset DM and three cases of exacerbations of DM following SARS-CoV-2 vaccination among a series of 53 DM patients. We discuss the potential causal relationship between vaccination and DM.

## Materials and methods

### Patient recruitment

All patients recruited in our study had a diagnosis of DM, and met the 2017 ACR/EULAR classification criteria for DM (14). All patients seen in the Divisions of Rheumatology and Dermatology with a pre-existing diagnosis of DM or a new diagnosis of DM were invited to the study. This included all patients in our catchment zone, with a population of 2 million. An ethics application was approved by the Research and Ethics Board at the University of Alberta (Pro00116853) in compliance with the Declaration of Helsinki. Consent was obtained from all participants included in this study. A total of 53 patients were contacted and responded to our study. 43 patients did not respond or could not be contacted, and 5 patients were deceased. Phone interviews using predetermined questionnaires were conducted to determine self-reported vaccination status and subjective symptoms associated with disease exacerbation (Supplementary material 1).

### Determination of disease exacerbation

Disease exacerbation or “flare” was defined as at least two or more of the following: increased fatigue, increased muscle soreness and/or weakness, and development or worsening of cutaneous findings in dermatomyositis such as heliotrope rash or Gottron’s papules. Although the predetermined questionnaire inquired about onset of subjective symptoms within 1 week of vaccination, the accepted time period for vaccine-associated DM exacerbation was 0–30 days. Any patients who subjectively reported disease exacerbation received a follow-up unscripted phone interview from MO or AC to further clarify their symptoms. These patients and their laboratory investigations were subsequently adjudicated by three members of the study team (AC, MO, RG). Consensus was reached by discussion.

### Data elements extracted

Age, sex, autoantibodies (which included myositis specific autoantibodies, anti-Mi-2, TIF-1γ, NXP-2, Jo-1, PL-7, PL-12, and MDA-5), immunomodulatory therapies prior to vaccination and in response to increased disease activity, and creatine kinase (CK) levels up to 1 year following the first dose of vaccination were collected from the electronic medical record (EMR) for all patients. Vaccination status and dates were obtained from patients directly *via* phone interviews, then confirmed using EMR for all patients. For patients with a notable vaccine-associated DM flare, symptoms and extent of organ involvement as per Myositis Intention to Treat Activity Index (MITAX) (15) were extracted, along with subsequent addition of immunomodulatory therapies.

## Results

The demographics of our cohort are summarized in Table 1. Of the 53 participants, 46 were female (86.8%) and 7 were male (13.2%). The mean age was 57.4 years, with a range of 18–89 years. The majority of patients with dermatomyositis in our cohort did not report disease exacerbation (90.6%). Eight patients were found to have CK elevations despite being asymptomatic (15.1%). These CK elevations occurred within 1 year following the first dose of vaccination during their routine DM follow-ups. All elevated CK values were found to be less than 1.5 times the upper limit of normal.

**TABLE 1:** Demographic summary of all dermatomyositis patients.

Patient demographics		
Total N patients		53
Sex (N; %)	Female	46 (86.8%)
	Male	7 (13.2%)
Mean age (Mean; Range)		57.4 (18–89)
Elevated creatine kinase without reported flare (>198U/L) (N; %)		8 (15.1%)
Mean vaccine doses (Mean; Range)		2.62 (1–3)
	Dose mixing	5 (9.43%)
	No dose mixing	45 (84.9%)
	Vaccine not specified^a^	3 (5.66%)
Mean number of drugs used for immunosuppression (Range)		1.51 (0–3)
	No immunosuppression (N; %)	5 (9.43%)
	Single agent (N; %)	22 (41.5%)
	Two agents (N; %)	20 (37.7%)
	Three agents (N; %)	6 (11.3%)
Immunosuppression regimen (excluding prednisone)	Hydroxychloroquine (N; %)	20 (37.7%)
	Methotrexate (N; %)	20 (37.7%)
	Azathioprine (N; %)	4 (7.55%)
	Mycophenolate mofetil (N; %)	8 (15.1%)
	Intravenous immunoglobulins (N; %)	20 (37.7%)
	Subcutaneous immunoglobulins (N; %)	5 (9.43%)
	Rituximab (N; %)	3 (5.66%)

^a^vaccine records not found in provincial vaccine registry due to residence in different province but patients self-reported minimum one dose in survey.

Of 51 patients with existing diagnoses of DM, three patients (5.88%) experienced DM symptom flares following vaccination (Table 2) with a mean MITAX score of 4.3 indicating mild disease. In addition, two patients received a new diagnosis of dermatomyositis, of which one patient required hospitalization, with a mean MITAX score of 18.5. Reported onset of disease ranged from 1 to 30 days, with a mean of 12.8 days. The majority of patients experiencing a flare did not require changes in immunomodulation therapy (median MITAX score 5; mean MITAX score 4.3). Overall disease activity was low in all five patients with a mean MITAX score of 10.

**TABLE 2:** Summary of patient experiencing dermatomyositis exacerbation after vaccination.

Patient (age/sex)	Onset of symptoms after vaccination	Vaccine, dose #	Clinical findings	Organ involvement	Autoantibodies	MITAX score
1 (53F)	30 days	AstraZeneca, dose 1	Initially presented with R flank pain due to PE, but later developed myalgia, joint pain, Gottron’s sign, Mechanic’s Hand, holster sign, shawl sign, V-neck sign, ragged cuticles, capillary loop dilation	Myocarditis (peak troponin 133 ng/L) Pericarditis Interstitial lung disease (usual interstitial pneumonia pattern) Muscle weakness, positive biopsy and MRI findings (normal CK) Venous thrombosis	ANA+, ENA+ RF+ anti-Ro-52+ SSA+ PL-12+	26
2 (76F)	24 days	Pfizer-BioNTech, dose 2	Fatigue, heliotrope rash, Gottron’s papules, shawl sign, periungual erythema with capillary loop dilation, ragged cuticles, panniculitis with skin ulcerations	Proximal muscle weakness, normal CK	ANA+ SAE-1+	11
3 (49F)	2 days	Pfizer-BioNTech, dose 2	Heliotrope rash, Gottron’s papules, periungual capillary loop dilation, cuticle hemorrhages	Proximal muscle weakness, normal CK	TIF-1γ+ PM/Scl100+ PM/Scl75+	5
4 (36F)	1 day	Moderna, dose 2	Ill-defined erythema of cheeks, V-neck sign, fatigue, myalgia	Muscle weakness, normal CK	Mi-2+	3
5 (79F)	7 days	Pfizer-BioNTech, dose 2	Shawl sign, Gottron’s papules, holster sign, fatigue, myalgia	Distal muscle weakness, normal CK	ANA+ Negative myositis panel	5

On average, participants received 2.62 vaccinations against SARS-CoV-2, with a range of one to three doses at the end of the study period. A total of 84.9% of patients had no dose mixing. A total of 9.43% of patients had mixed doses. Three patients (5.66%) self-reported having received at least one vaccination dose of Pfizer-BioNTech; however, the records could not be confirmed with the provincial vaccine registry due to limitations in the EMR for these particular patients.

For patients with historical DM, a majority of them were on combination immunomodulatory therapies with the most common therapies including hydroxychloroquine (37.7%), methotrexate (37.7%), and intravenous immunoglobulins (37.7%).

We have summarized the specific case summaries for the patients with a new diagnosis of DM (cases 1 and 2), and those with a disease-associated flare (cases 3, 4, and 5).

### Case summaries

Patient 1 presented with internal iliac vein thrombosis and bilateral basilar pulmonary emboli, accompanied by wedge shaped consolidative opacity at the right base, approximately 4 weeks after receiving COVAUVec vaccine. This was treated with rivaroxaban. Approximately 5 weeks after anticoagulation, the patient presented with fever, joint pain (elbows, wrists, hands, knees, and ankles), and worsening shortness of breath. Troponin and C-reactive protein (CRP) were elevated, and the patient was positive for SSA antibodies (MITAX score 26). Positron emission tomography (PET) scan showed bilateral scattered consolidative opacities more prominent at the lung bases, along with borderline enhanced mediastinal, hilar, and axillary lymphadenopathy. The patient was started on prednisone 40 mg daily, methotrexate, and hydroxychloroquine for presumed Sjogren’s disease. However, as prednisone was tapered, she developed a skin rash, fever, severe dyspnea and chest pain, and was rehospitalized 6 months later. CT scan showed evolution of the previous lung lesions to basal-predominant subpleural reticulation with associated bronchiectasis/bronchiolectasis, showing the pattern of usual interstitial pneumonia (UIP) or non-specific interstitial pneumonia (NSIP). Hospital stay was complicated by atrioventricular nodal reentry tachycardia requiring rate control with amiodarone and diltiazem, and cardiac MRI showed pericarditis. Diagnosis of dermatomyositis (with concomitant pericarditis and interstitial lung disease) was made based on skin findings, PL-12 autoantibodies, and a positive muscle biopsy (vastus medialis). Treatment with prednisone and mycophenolate mofetil (MMF) led to an improvement of her symptoms. This was followed by a course of intravenous immunoglobulins (IVIG).

Patient 2, a previously healthy female, developed fatigue, voice hoarseness and skin lesions on the arms, trunk, and face, 3 weeks after her second dose of the Pfizer-BioNTech vaccine. On examination, she presented with proximal muscle weakness and skin signs of dermatomyositis (MITAX 11). Serology for anti-SAE-1 was positive. Nailfold video capillaroscopy revealed severe capillary disorganization with capillary enlargement and microhemorrhages. She initially did not respond to prednisone monotherapy, but disease improvement was achieved with the use of MMF and IVIG.

Patient 3 had a previous history of TIF-1γ positive DM and was in complete remission for a period of 2 years on hydroxychloroquine. After receiving the second dose of the Pfizer-BioNTech vaccine, she developed a heliotrope rash and prominent muscle weakness which spontaneously improved within 2 weeks (MITAX 5). We maintained watchful observation, but during the following month the cutaneous lesions flared, and she developed proximal muscle weakness. The patient declined systemic steroids, and IVIG was initiated.

Patient 4 had a previous history of anti-Mi-2 DM that was in full remission for the last 18 months, maintained on azathioprine, hydroxychloroquine, and monthly infusions of methylprednisolone. One day following her second dose of the Moderna vaccine, she developed ill-defined erythema on her cheeks, V-neck sign, and mild myalgia which spontaneously improved over 2 days (MITAX 3). She did not require any change in medications and had not developed symptoms with her first dose of Moderna, or her third dose of Pfizer-BioNTech.

Patient 5 had a previous history of inclusion body myositis which was relatively quiescent, diagnosed by muscle biopsy in August 2019. Within 1 week of her first dose of the Pfizer-BioNTech vaccine, she developed shawl sign and Gottron’s papules, with no myalgia. She received her second dose of Pfizer-BioNTech 4 weeks after the first dose, and noted mild myalgia, and worsening of the shawl sign and Gottron’s papules. Her skin findings continued to progress to include her scalp and legs, until she was diagnosed with dermatomyositis/inclusion body myositis overlap syndrome based on complement staining on skin biopsy in August 2021, and subtle perimysial pathology on repeat muscle biopsy. After her third dose of Pfizer-BioNTech in November 2021, she noted significant worsening of fatigue, myalgia, and her skin rashes (MITAX 5). She did not have CK elevation despite her myalgias. Throughout these reactions, she remained on IVIG during all three doses; she had been unable to tolerate prednisone, methotrexate, and mycophenolate mofetil in the past.

## Discussion

We have observed two patients who have new presentations and three patients with exacerbations of existing DM following receiving a vaccine for SARS-CoV-2. This represents a small but significant proportion of patient series (9.8%) compared to the general population which has been reported to have 0.6% prevalence of adverse events overall in Canada (16). There was no association between the presence of myositis specific autoantibodies, or elevations in CK with DM flares in our patient series.

The incidence of DM exacerbation before and after the onset of COVID-19 is difficult to compare as the literature is limited and definitions for disease exacerbations are not standardized. However, the incidence of disease exacerbation in our case series is low in comparison to one survey of 524 patients from 2018 to 2019 on patient-reported exacerbation in dermatomyositis and polymyositis, where 378 patients (72.1%) reported at least one flare in the past 12 months (17). One thought is that COVID-19 and telemedicine has created challenges in monitoring patient disease activity and adapting immunosuppression regimens, although this would likely lead to an increased frequency of relapses, rather than a lower incidence.

We acknowledge certain limitations in our methodology as well. Approximately 50% of patients were unable to be reached by telephone, which introduces the possibility of selection bias. There was also a small number of patients who were deceased (*n* = 5) at the time of the questionnaires; the cause of death for patients was unknown, and a relationship to vaccination cannot be excluded as their records were not reviewed. Recall bias is another limitation as patients may not have remembered their symptoms, and symptoms of exacerbation are subjective. Our questionnaire was conducted from November to December of 2021. First dose vaccination was available to the general public in Canada around April 2021, however, each individual patients’ first doses varied between January 2021 to December 2021. For this reason, the CK values up to 1 year post-vaccination were reviewed during data extraction in January 2022 to evaluate for possible trends or asymptomatic elevation. We also acknowledge the limitations of asking patients about symptoms of exacerbations in a closed-ended format and within a restricted short time period of 1 week, as this may also have led to under-reporting, although the patients that experienced vaccine-associated flares were closely followed by MO and RG, and frequently interacted with them.

There have been several other reports of dermatomyositis and idiopathic inflammatory myopathies developing or relapsing after SARS-CoV-2 vaccination, which supports our findings (18–22). Overall it is difficult to conclude whether there is a true correlation between dermatomyositis exacerbation and vaccination, however, there is emerging literature describing several mechanisms that propose how vaccinations may promote the development of inflammatory diseases such as DM. DM has been associated with infectious triggers, but it has also been suggested that *de novo* diagnosis of DM following COVID-19 infection may be a mimic of COVID-19 myositis (12, 13). Initial case reports of myositis following active COVID-19 infection discussed the possibility of direct virus entry into muscle *via* ACE-2, viral trigger of innate immune activation or autoinflammation, and viral adaptive immune activation (23). Similar phenomena of vaccine-induced autoimmune disease have been reported with viral RNA from the influenza vaccine contributing to the development of ANCA-vasculitis (24). Autoimmune/inflammatory syndrome in response to adjuvants (ASIA) syndrome has also been reported after SARS-CoV-2 vaccination (25, 26).

It is well established that patients with connective tissue diseases such as systemic lupus erythematosus (SLE) and DM develop elevated type I interferon responses in the prodromal phases anteceding clinical manifestations of their disease (27, 28). These patients are more sensitive to flares in their disease resulting from viral infections. In SLE, the presence of these signals is associated with pattern recognition receptors such as toll-like receptor 7 (TLR7) which are known to recognize ssRNA. Indeed, it has recently been suggested that patients with SLE may have increased frequencies of gain of function mutations in TLR7 which are sufficient to promote inflammatory manifestations associated with SLE such as chronic B cell activation (29). This may explain why several groups have reported new diagnoses of SLE or severe SLE flares after COVID-19 vaccinations as we have described for DM (30–33). As TLR7 plays a prominent role in the pathogenesis of DM (34), we suspect that TLR7 agonists such as those present in RNA/DNA vaccines utilized for COVID-19 may be sufficient to promote inflammatory signals associated with DM. Another possibility may include a role for vaccine encoded spike proteins promoting a “superantigen” response, which promotes systemic immune dysregulatory responses in genetically predisposed individuals, as previously suggested (35). Of note, a study of vaccinated DM and SLE patients demonstrated that more fully vaccinated dermatomyositis patients had disease exacerbation following the vaccine compared to their SLE counterparts, the reason for which is unknown but may be related to the role of plasmacytoid dendritic cells in SLE (36).

We speculate that one potentially protective mechanism for patients with DM and/or SLE is the use of TLR7 inhibitory anti-rheumatic therapies such as antimalarial therapies (e.g., hydroxychloroquine or chloroquine). These agents are known to attenuate TLR7 signals through a variety of mechanisms (37). This observation is supported in our cohort where a large number (37.7%) of patients were using an antimalarial agent. Similarly, in patients with established SLE (many of which were on hydroxychloroquine) treated with a SARS-CoV-2 vaccine, the rates of adverse reactions were low, which may be explained in part by the use of these agents (31). Further to this, most of the patients in our cohort with adequate disease control using combination immunomodulatory agents did not experience significant adverse reactions from vaccination–suggesting a role for immunomodulation in quelling vaccine related inflammatory responses in patients with DM. In addition, in the patients with a disease related flare that were using an antimalarial agent, their flares were mild and self-limited compared to the more severe ones noted in patients not using an antimalarial agent.

Finally, an intriguing theory may suggest a role for molecular mimicry as a potential cause for vaccine-associated flares in DM patients. Megremis et al. (38) reported three immunogenic linear epitopes with high sequence identity to SARS-CoV-2 proteins in patients with autoimmune DM. Based on these findings that viral spike glycoproteins share amino acid sequences with DM autoantigens, we consider it plausible that molecular mimicry to viral proteins in the vaccine in the presence of RNA serving as adjuvants may promote exacerbations in DM. These results may be extrapolated to post-vaccine myocarditis (4, 39, 40).

## Conclusion

Our observations suggest a possible correlation between SARS-CoV-2 vaccination and exacerbation of dermatomyositis. There are no current studies with appropriate design to establish a relationship between vaccination and disease exacerbation. However, there are several emerging pathophysiological arguments that support this hypothesis including that spike (S) protein-coding SARS-CoV-2 vaccines may induce or exacerbate dermatomyositis, analogously to what was shown in patients with COVID-19 disease. Most of the patients with disease-associated flares experienced proximal muscle weakness (with normal CK values) and typical cutaneous findings and nailfold capillary changes described in DM. DM-related flares can result in cardiac and/or pulmonary involvement which is important to distinguish from COVID-19 vaccine-induced myocarditis, pericarditis and interstitial lung disease. The mechanism of vaccine-induced dermatomyositis remains unknown although we speculate that it may be promoted by aberrant TLR7 signaling, and possible molecular mimicry between viral spike glycoproteins, anti-idiotype antibodies, and dermatomyositis autoantigens.

## Data availability statement

The original contributions presented in this study are included in the article/Supplementary material, further inquiries can be directed to the corresponding author/s.

## Ethics statement

The studies involving human participants were reviewed and approved by Research and Ethics Board at the University of Alberta (Pro00116853). The patients/participants provided their written informed consent to participate in this study. Written informed consent was obtained from the individual(s) for the publication of any potentially identifiable images or data included in this article.

## Author contributions

AC: literature search, study design, data collection, data analysis, and writing. JC, EY, GF, and PH: review and editing. DR: data collection. MO and RG: study design and writing. All authors contributed to the article and approved the submitted version.

## References

[1] Government of Canada,. *Health InfoBase COVID-19 Vaccination Coverage.* (2022). Available online at: https://health-infobase.canada.ca/covid-19/vaccination-coverage/ (accessed May 1, 2022).

[2] Ferri C Giuggioli D Raimondo V L’Andolina M Tavoni A Cecchetti R COVID-19 and rheumatic autoimmune systemic diseases: report of a large Italian patients series. *Clin Rheumatol.* (2020) 39:3195–204. 10.1007/s10067-020-05334-7 32852623 PMC7450255

[3] Government of Canada. *Reported Side Effects Following COVID-19 Vaccination in Canada.* (2022). Available online at: https://health-infobase.canada.ca/covid-19/vaccine-safety/#a6 (accessed April 29, 2022).

[4] Witberg G Barda N Hoss S Richter I Wiessman M Aviv Y Myocarditis after Covid-19 vaccination in a large health care organization. *N Engl J Med.* (2021) 385:2132–9. 10.1056/NEJMoa2110737 34614329 PMC8531986

[5] Dourmishev L Guleva D Pozharashka J Drenovska K Miteva L Vassileva S. Autoimmune connective tissue diseases in the COVID-19 pandemic. *Clin Dermatol.* (2021) 39:56–63. 10.1016/j.clindermatol.2020.12.013 33972054 PMC7833035

[6] Borges NH Godoy TM Kahlow BS. Onset of dermatomyositis in close association with COVID-19-a first case reported. *Rheumatology.* (2021) 60:SI96. 10.1093/rheumatology/keab290 33757122 PMC8083658

[7] Gokhale Y Patankar A Holla U Shilke M Kalekar L Karnik ND Dermatomyositis during COVID-19 pandemic (a case series): is there a cause effect relationship? *J Assoc Physicians India.* (2020) 68:20–4. 33187031

[8] Ho BVK Seger EW Kollmann K Rajpara A. Dermatomyositis in a COVID-19 positive patient. *JAAD Case Rep.* (2021) 13:97–9. 10.1016/j.jdcr.2021.04.036 34056056 PMC8149169

[9] Liquidano-Perez E García-Romero MT Yamazaki-Nakashimada M Maza-Morales M Rivas-Calderón MK Bayardo-Gutierrez B Juvenile dermatomyositis triggered by SARS-CoV-2. *Pediatr Neurol.* (2021) 121:26–7. 10.1016/j.pediatrneurol.2021.05.011 34126319 PMC8136292

[10] Rodero MP Pelleau S Welfringer-Morin A Duffy D Melki I Bader-Meunier B Onset and relapse of juvenile dermatomyositis following asymptomatic SARS-CoV-2 infection. *J Clin Immunol.* (2022) 42:25–27. 10.1007/s10875-021-01119-y 34426906 PMC8382211

[11] Shahidi Dadras M Rakhshan A Ahmadzadeh A Hosseini SA Diab R Safari Giv T Dermatomyositis-lupus overlap syndrome complicated with cardiomyopathy after SARS-CoV-2 infection: a new potential trigger for musculoskeletal autoimmune disease development. *Clin Case Rep.* (2021) 9:e04931. 10.1002/ccr3.4931 34667608 PMC8512177

[12] Tanboon J Nishino I. COVID-19-associated myositis may be dermatomyositis. *Muscle Nerve.* (2021) 63:E9–10. 10.1002/mus.27105 33095493

[13] Movahedi N Ziaee V. COVID-19 and myositis; true dermatomyositis or prolonged post viral myositis? *Pediatr Rheumatol Online J.* (2021) 19:86. 10.1186/s12969-021-00570-w 34112199 PMC8190732

[14] Lundberg IE Tjärnlund A Bottai M Werth VP Pilkington C Visser M 2017 European League Against Rheumatism/American College of Rheumatology classification criteria for adult and juvenile idiopathic inflammatory myopathies and their major subgroups. *Ann Rheum Dis.* (2017) 76:1955–64. 10.1136/annrheumdis-2017-212786 29079590 PMC5736307

[15] Isenberg DA Allen E Farewell V Ehrenstein MR Hanna MG Lundberg IE International consensus outcome measures for patients with idiopathic inflammatory myopathies. Development and initial validation of myositis activity and damage indices in patients with adult onset disease. *Rheumatology.* (2004) 43:49–54. 10.1093/rheumatology/keg427 12867580

[16] Public Health Agency of Canada. *COVID-19 Vaccine Safety: Summary of Weekly Report on Side Effects Following Immunization – Canada.CA.* (2021). Available online at: https://health-infobase.canada.ca/covid-19/vaccine-safety/summary.html (accessed June 28, 2022).

[17] Christopher-Stine L Wan GJ Kelly W McGowan M Bostic R Reed ML. Patient-reported dermatomyositis and polymyositis flare symptoms are associated with disability, productivity loss, and health care resource use. *J Manag Care Spec Pharm.* (2020) 26:1424–33. 10.18553/jmcp.2020.26.11.1424 33119444 PMC10391285

[18] De Marco G Giryes S Williams K Alcorn N Slade M Fitton J A large cluster of new onset autoimmune myositis in the yorkshire region following SARS-CoV-2 vaccination. *Vaccines.* (2022) 10:1184. 10.3390/vaccines10081184 35893834 PMC9331977

[19] Conticini E d’Alessandro M Grazzini S Fornaro M Sabella D Lopalco G Relapses of idiopathic inflammatory myopathies after vaccination against COVID-19: a real-life multicenter Italian study. *Intern Emerg Med.* (2022) 17:1921–8. 10.1007/s11739-022-03028-3 35754076 PMC9244457

[20] Camargo Coronel A Jiménez Balderas FJ Quiñones Moya H Hernández Zavala MR Mandinabeitia Rodríguez P Hernández Vázquez JR Dermatomyositis post vaccine against SARS-COV2. *BMC Rheumatol.* (2022) 6:20. 10.1186/s41927-022-00250-6 35361289 PMC8970647

[21] Chaima K Mariem A Sana B Khadija S Mariem R Massara B Vaccine-induced dermatomyositis following COVID-19 vaccination. *Dermatol Ther.* (2022) 35:e15749. 10.1111/dth.15749 35906839 PMC9353435

[22] Venkateswaran K Aw DC-W Huang J Angkodjojo S. Dermatomyositis following COVID-19 vaccination. *Dermatol Ther.* (2022) 35:e15479. 10.1111/dth.15479 35355380 PMC9111860

[23] Saud A Naveen R Aggarwal R Gupta L. COVID-19 and myositis: what we know so far. *Curr Rheumatol Rep.* (2021) 23:63. 10.1007/s11926-021-01023-9 34216297 PMC8254439

[24] Jeffs LS Nitschke J Tervaert JWC Peh CA Hurtado PR. Viral RNA in the influenza vaccine may have contributed to the development of ANCA-associated vasculitis in a patient following immunisation. *Clin Rheumatol.* (2016) 35:943–51. 10.1007/s10067-015-3073-0 26361945

[25] Das L Bhadada SK Sood A. Post-COVID-vaccine autoimmune/inflammatory syndrome in response to adjuvants (ASIA syndrome) manifesting as subacute thyroiditis. *J Endocrinol Invest.* (2022) 45:465–7. 10.1007/s40618-021-01681-7 34585363 PMC8478264

[26] Jara LJ Vera-Lastra O Mahroum N Pineda C Shoenfeld Y. Autoimmune post-COVID vaccine syndromes: does the spectrum of autoimmune/inflammatory syndrome expand? *Clin Rheumatol.* (2022) 41:1603–9. 10.1007/s10067-022-06149-4 35378658 PMC8979721

[27] Greenberg SA. Dermatomyositis and type 1 interferons. *Curr Rheumatol Rep.* (2010) 12:198–203. 10.1007/s11926-010-0101-6 20425524 PMC2929916

[28] Crow MK. Type I interferon in the pathogenesis of lupus. *J Immunol.* (2014) 192:5459–68. 10.4049/jimmunol.1002795 24907379 PMC4083591

[29] Brown GJ Cañete PF Wang H Medhavy A Bones J Roco JA TLR7 gain-of-function genetic variation causes human lupus. *Nature.* (2022) 605:349–56. 10.1038/s41586-022-04642-z 35477763 PMC9095492

[30] Barbhaiya M Levine JM Siegel CH Bykerk VP Jannat-Khah D Mandl LA. Adverse events and disease flares after SARS-CoV-2 vaccination in patients with systemic lupus erythematosus. *Clin Rheumatol.* (2022) 41:1619–22. 34716843 10.1007/s10067-021-05963-6 PMC8556788

[31] Zavala-Flores E Salcedo-Matienzo J Quiroz-Alva A Berrocal-Kasay A. Side effects and flares risk after SARS-CoV-2 vaccination in patients with systemic lupus erythematosus. *Clin Rheumatol.* (2022) 41:1349–57. 34782941 10.1007/s10067-021-05980-5 PMC8592807

[32] Kaur I Zafar S Capitle E Khianey R. COVID-19 vaccination as a potential trigger for new-onset systemic lupus erythematosus. *Cureus.* (2022) 14:e21917. 10.7759/cureus.21917 PMC8901143 35273863

[33] Molina-Rios S Rojas-Martinez R Estévez-Ramirez GM Medina YF. Systemic lupus erythematosus and antiphospholipid syndrome after COVID-19 vaccination. A case report. *Mod Rheumatol Case Rep.* (2022). 1–4. 10.1093/mrcr/rxac018 35246682 PMC8903504

[34] Meyer A Alsaleh G Heuschling C Gottenberg JE Georgel P Geny B Dermatomyositis flare on imiquimod therapy highlights a crucial role of aberrant TLR7 signalling. *RMD Open.* (2016) 2:e000294. 10.1136/rmdopen-2016-000294 27933205 PMC5133400

[35] Cheng MH Zhang S Porritt RA Noval Rivas M Paschold L Willscher E Superantigenic character of an insert unique to SARS-CoV-2 spike supported by skewed TCR repertoire in patients with hyperinflammation. *Proc Natl Acad Sci U.S.A.* (2020) 117:25254–62. 10.1073/pnas.2010722117 32989130 PMC7568239

[36] Sprow G Afarideh M Dan J Feng R Keyes E Grinnell M Autoimmune skin disease exacerbations following COVID-19 vaccination. *Front Immunol.* (2022) 13:899526. 10.3389/fimmu.2022.899526 35693768 PMC9186119

[37] Kuznik A Bencina M Svajger U Jeras M Rozman B Jerala R. Mechanism of endosomal TLR inhibition by antimalarial drugs and imidazoquinolines. *J Immunol.* (2011) 186:4794–804. 10.4049/jimmunol.1000702 21398612

[38] Megremis S Walker TDJ He X Ollier WER Chinoy H Hampson L Antibodies against immunogenic epitopes with high sequence identity to SARS-CoV-2 in patients with autoimmune dermatomyositis. *Ann Rheum Dis.* (2020) 79:1383–6. 10.1136/annrheumdis-2020-217522 32444414 PMC7509518

[39] Mevorach D Anis E Cedar N Bromberg M Haas EJ Nadir E Myocarditis after BNT162b2 mRNA vaccine against Covid-19 in Israel. *N Engl J Med.* (2021) 385:2140–9. 34614328 10.1056/NEJMoa2109730 PMC8531987

[40] Bozkurt B Kamat I Hotez PJ. Myocarditis with COVID-19 mRNA vaccines. *Circulation.* (2021) 144:471–84. 34281357 10.1161/CIRCULATIONAHA.121.056135 PMC8340726

