# Supplementary material for: A case series of dermatomyositis following SARS-CoV-2 vaccination
Source: Front Med (Lausanne). 2022 Nov 7;9:1013378. doi: 10.3389/fmed.2022.1013378 (PMC9676274; doi:10.3389/fmed.2022.1013378)
Supplement: Supplementary file 1 [file Data_Sheet_1.pdf]

Supplementary Material

S1. Predetermined Questionnaire for Patient Phone Interviews

1. Did you receive the COVID Vaccine?
2. What vaccine did you receive and how many doses of each?
3. Did you have a reaction to the vaccine?
4. Did it cause a flare up of your dermatomyositis?
5. Did you have flu-like symptoms up to 1 week after your vaccine?
